# Animal Models of Lassa Fever

**DOI:** 10.3390/pathogens9030197

**Published:** 2020-03-06

**Authors:** Rachel A. Sattler, Slobodan Paessler, Hinh Ly, Cheng Huang

**Affiliations:** 1 Department of Pathology, University of Texas Medical Branch, 301 University Blvd., Galveston, TX 77555-0609, USA; rasattle@utmb.edu (R.A.S.); slpaessl@utmb.edu (S.P.); 2 Department of Veterinary Biomedical Sciences, University of Minnesota, Twin Cities, 1988 Fitch Ave., 295H Animal Science Veterinary Medicine Bldg., Saint Paul, MN 55108, USA; hly@umn.edu

**Keywords:** arenaviruses, Lassa virus, viral hemorrhagic fevers, Lassa fever, animal models

## Abstract

Lassa virus (LASV), the causative agent of Lassa fever, is estimated to be responsible for up to 300,000 new infections and 5000 deaths each year across Western Africa. The most recent 2018 and 2019 Nigerian outbreaks featured alarmingly high fatality rates of up to 25.4%. In addition to the severity and high fatality of the disease, a significant population of survivors suffer from long-term sequelae, such as sensorineural hearing loss, resulting in a huge socioeconomic burden in endemic regions. There are no Food and Drug Administration (FDA)-approved vaccines, and therapeutics remain extremely limited for Lassa fever. Development of countermeasures depends on relevant animal models that can develop a disease strongly mimicking the pathogenic features of Lassa fever in humans. The objective of this review is to evaluate the currently available animal models for LASV infection with an emphasis on their pathogenic and histologic characteristics as well as recent advances in the development of a suitable rodent model. This information may facilitate the development of an improved animal model for understanding disease pathogenesis of Lassa fever and for vaccine or antiviral testing.

## 1. Introduction

Lassa virus (LASV) is the causative agent of Lassa fever (LF). The virus was first isolated from two missionary nurses who died from LF in Nigeria in 1969 [1,2,3]. A third nurse was infected and brought to the United States; her plasma was used to treat a fourth patient who was exposed by handling infected blood samples [1]. LASV is a mammarenavirus from the *Arenaviridae* family and has a single-stranded, negative-sense, bisegmented RNA genome. LASV is endemic throughout Western Africa, especially in Nigeria, Liberia, Guinea, and Sierra Leone, where it is transmitted by the Natal mastomys (*Mastomys natalensis*) rodent, although recent work suggests viral presence in the reddish-white mastomys (*Mastomys erythroleucus*) and the “African wood mouse” (*Hylomyscus pamfi*) rodents as well [2,4]. LASV isolates of different geographic and host origins are highly diverse in genomic sequences and phylogenetically classified into up to seven lineages, with each lineage predominately localized in specific countries (refer to Table 1 for the LASV isolates covered in this review) [5,6,7,8,9]. A recent deep sequencing analysis revealed that the sequence variations could be as high as 25% and 32% in the viral genomic small (S) and large (L) segments, respectively, among different strains [5]. LASV causes zoonotic infections in humans, typically through the ingestion of contaminated food and/or water or inhalation of tainted aerosols from infected rodents; person-to-person transmission occurs via contact with bodily fluids [2]. Estimates from the Centers for Disease Control and Prevention (CDC) place the number of new LASV infections each year as high as 300,000 with around 5000 deaths from LF [2,10]. The average case fatality rate is approximately 1%, which can dramatically elevate to 15–20% for hospitalized cases [2]. However, a more recent report indicated only 633 confirmed cases and 171 deaths in Nigeria in 2018, with a 27% case fatality rate [11]. In the recent 2018–2019 Nigerian LF outbreaks, approximately 25% of patients died from the infection [12,13,14]. Therefore, it is necessary to re-evaluate the annual incidence of LASV infections and deaths.

There are no FDA-approved vaccines against LASV. Current clinical treatments are limited to an off-label use of ribavirin [2,14]. However, ribavirin treatment is expensive and only effective if administered within the first six days after the onset of symptoms [15]. When considering the high rate of misdiagnosis of LF for other endemic infections, such as typhoid or falciparum malaria [1,2,16,17], this time frame for effective LF treatment is extremely challenging [16]. Moreover, ribavirin has a high rate of side effects that often require further medical intervention, such as hemolysis [15]. Development of countermeasures against LF requires relevant animal models that can recapitulate disease and pathogenic features of LF in humans. The objective of this review is to survey the current Lassa fever animal models, including their pathologic and histologic characteristics as well as new advances in small rodent models, in order to inform the development of new animal models. 

## 2. Lassa Fever Symptoms and Pathogenesis

LF symptoms typically appear 1–3 weeks post-infection [2]. Eighty percent of cases are asymptomatic or present with mild, non-specific febrile symptoms such as fever, sore throat, headache, and general malaise, that may be misdiagnosed as typhoid, malaria, or appendicitis [1,2,16,17]. The other 20% of cases progress to much more severe symptomology [2]. These clinical manifestations include diarrhea, hemorrhage, disorientation, respiratory distress, abdominal pain, vomiting, facial edema, and severe pharyngitis [1,2]. Neurological signs such as hearing loss, encephalitis, and tremors also occur [2]. Of note, this hearing loss manifests as sudden-onset sensorineural hearing loss (SNHL) in approximately one-third of patients and is irreversible in two-thirds of those cases [10,18,19]. Infections during pregnancy have a greater risk of death as well as spontaneous abortion [20]. Death will typically occur due to multiorgan failure approximately two weeks post-onset of symptoms [2]. The long-term sequelae, such as SNHL, are generating a huge socioeconomic burden across Western Africa, where stigmatization and isolation lead to increased rates of depression and unemployment [21]. Aid programs in Nigeria alone can cost upwards of $43 million each year [18].

Clinical laboratory findings include thrombocytopenia, leucopenia with lymphopenia, elevated blood urea and proteinuria [22]. The aspartate aminotransferase (AST), alanine aminotransferase (ALT), amylase, and creatine phosphokinase (CPK) concentrations are also elevated in patient’s serum [17], often with a higher AST level than ALT level, suggesting that the transaminases in serum are not solely derived from damaged liver, but may also be from other tissues [23]. High viral titers are measured in the spleen, lung, liver, kidney, heart, mammary gland, and placenta [17]. The level of viremia has been correlated with the severity of disease outcome.

Current knowledge on the pathology of Lassa fever is still limited due to civil unrest and social taboos and customs when dealing with the deceased in endemic areas [22]. Available pathologic analysis of clinical cases indicates lesions in the spleen, liver, and adrenal glands [17]. Histologic analysis of the liver indicates significant eosinophilic necrosis and parenchymal cell necrosis with an infiltration of eosinophils in the sinusoids [1,17,24]. Spleen samples indicate a presence of eosinophilic necrosis, lymphoid depletion, atrophied white pulp, and fibrin deposits with an infiltration of lymphocytes and mononuclear cells [1,17,24]. Adrenal gland samples indicate multifocal adrenocortical cellular necrosis, often associated with inflammation [17]. Less frequent findings include petechiae of the gastrointestinal tract, interstitial nephritis, renal tubular injury, lymph node sinus histiocytosis, mild interstitial pneumonia, renal edema and hemorrhage, as well as mild interstitial mononuclear myocarditis [1,17,24]. No alterations are detected in placental, mammary, uterine, ovarian, pancreatic, central nervous system (CNS), or brain samples [17]. These observations were made from a study performed in 1982. Our knowledge on pathology of LF in humans still lack. Studies with non-human primates have provided valuable information regarding the pathogenesis of LASV infection. As there is a limitation in study with non-human primates (NHPs), a small animal model that can resemble pathological changes in LF patients is desired.

## 3. Murine Models

### 3.1. Natal Mastomys (Mastomys Natalensis) Mice

Natal mastomys mice are the natural host of LASV. The exact subspecies that acts as a LASV reservoir remains unclear, due to multiple subspecies coexisting in endemic regions and their high genetic diversity [25,26]. There are very few studies on laboratory infections of Natal mastomys due to lack of laboratory clone of these mice. One early study reported that LASV caused a chronic asymptomatic infection despite the high virus titers detected in organs at 74 days post-infection (dpi) [25] (Table 2). Affected tissues included the lung, spleen, liver, kidney, brain, bladder, and lymph nodes [25]. Virus was also detected in the blood and urine. As the Natal mastomys mice did not develop LF signs, its use in viral pathogenesis or vaccine studies is limited. However, this model may prove useful for basic transmission studies as well as studies developing methods for limiting transmission of the virus between rodent populations or from rodents to humans.

### 3.2. IFNAR^-/-^ Mice

Immune-competent laboratory mice are generally resistant to LASV infection [27,28,29,30]. Interferons play important roles in regulating host innate and adaptive immune responses against infections. LASV infection of the 129Sv mice lacking the receptor for interferon α and β (IFNAR^-/-^) results in a non-lethal acute infection accompanied with persistent viremia [27,28,29,30] (Table 2). Infected mice lose approximately 15% body weight but did not develop febrile or neurological signs [26,29]. Other disease signs included ruffled fur and hypoactivity occurring by 11 dpi [28,30]. Peak viremia occurred approximately 8 dpi. The lung, liver, and spleen had the highest viral loads, and virus was also present in the brain, kidney, and heart at much lower titers [27]. Similar pathological changes and disease signs were found in the animals upon infection with a variety of LASV strains, including the Josiah, AV, BA366, and Nig04-10 strains isolated from endemic countries, Sierra Leone, Côte d’Ivoire, Guinea, and Nigeria, respectively [27,28].

A chimeric IFNAR^-/-B6^ lethal mouse model had been established, in which the IFNAR^-/-^ mice were irradiated and transplanted with bone marrow progenitor cells from wild type C57BL/6 mice. These IFNAR^-/-B6^ chimeric mice uniformly succumbed to infections with a variety of LASV strains within 10 days [29,31]. LASV strains tested include Ba366, AV, Ba289, Nig04-10, and Nig-CSF, all of which were 100% lethal using 1000 focus-forming units (FFU) [29]. These mice presented with liver damage, vascular leakage, and systemic viral dissemination [29]. Virus titers were detected in the liver, lung, spleen, kidney, heart, brain, and blood [29,31]. Animals also presented with weight loss and hypothermia [31]. Moreover, this model featured an elevated AST/ALT ratio, as well as FAS and FAS-L [29,31] similarly observed in LF patients. Pathological findings included an infiltration of inflammatory leukocytes in the kidney, infiltration of granulocytes and T cell lymphocytes in the liver, and an increased presence of CD8+ T cells in both the spleen and lungs [29]. Lung and liver samples both presented with indications of significant vascular leakage as well as edema [29]. Interestingly, IFNAR^-/-B6^ mice with a depletion of CD8+ T cells exhibited significantly increased survival rate (87.5%) despite persistent viremia [29]. This depletion also reduced the FAS and FAS-L concentrations, as well as the vascular leakage in the liver and lung [29]. This model implicates the role of T cells in the pathogenesis of LF. IFNAR^-/-^ mice are less susceptible to disease probably as a result of lacking IFN receptors that are critical to stimulate a T cell response.

### 3.3. IFNαβ/γR^-/-^ Mice

The 129Sv mice lacking interferon α, β and γ receptors (IFNαβ/γR^-/-^) did not develop clinical signs of disease upon LASV infection apart from a minor and transient weight loss [30] (Table 2). Neither did this model develop sudden onset sensorineural hearing loss when infected with either the LF2384 or LF2350 clinical isolates from a fatal and a non-fatal case respectively, from the 2012 Sierra Leone outbreak [8]. These two clinical isolates have been passaged only once in Vero cells. The animals presented with less severe disease upon infection with the Josiah strain as IFNAR^-/-^ mice [30]. Virus was present in the serum, spleen, lung, liver, brain, and kidney, but was cleared by 25 dpi [30]. Tissue samples featured similar findings to the LASV-infected IFNAR^-/-^ model in the brain and lung, but to significantly lesser extents [30]. Liver samples presented with mononuclear cell infiltration, although inflammation was rarely observed [30]. In the spleen, only the red pulp was affected; the kidneys showed mild, regional nephritis [30].

### 3.4. STAT1^-/-^ Mice

Signal transducer and activator of transcription 1 (STAT1) is a transcription factor that is activated in response to IFN α, β, γ and IL-6. The 129Sv mice lacking STAT1 (STAT1^-/-^) were not only highly susceptible to LASV infection, including clinical isolates, but also presented with a lethal disease with clinically relevant manifestations, such as SNHL [8,28]. Thus, the STAT1^-/-^ mouse model is promising for vaccine and therapeutic testing. Mice intraperitoneally infected with 10^4^ plaque forming units (PFUs) of the Josiah strain of LASV lost ~18% of their body weight between 4 and 6 dpi, and either died or met humane endpoint criteria by 7 dpi [28]. This model also matched the differential clinical outcome of the patients from whom the viruses were isolated, with LF2384 infections being lethal and LF2350 infections non-lethal in these mice. Intraperitoneal (IP) injection with 10^5^ PFU of the LF2384 or the LF2350 isolates led to 80% and 10–50% lethality, respectively [8]. Infection with 10^4^ PFU of these viruses resulted in 80% lethality for the fatal isolate and 0% lethality for the non-fatal isolate [8]. LF2384 also caused fever and other signs of disease by 4–5 dpi, and death approximately 7 dpi [28]. Other signs of disease included weight loss, hypothermia, hunched posture, reduced activity, and ruffled fur [8]. In one study, fever was not noted, although the possibility that fever transiently occurred before turning into hypothermia was raised [8,28]. Virus was disseminated to the brain, liver, spleen, lung, kidney, and heart by 3 dpi with titers steadily increasing to their peak at 7 dpi [8,28]. Viremia appeared by 3 dpi and remained at high titers until death [28]. Increased ALT concentrations and decreased albumin, WBC, and monocyte counts were noted as early as 3 dpi; a moderate decrease in platelet counts was also observed [28]. Histologic analysis of the spleen indicated lesions with eosinophil accumulation in the red pulp, primarily focused around the periarteriolar lymphocytic sheaths as well as apoptotic and necrotic cells [28]. Samples also demonstrated significant fibrin deposits and an infiltration of macrophages filled with apoptotic nuclei [28]. Analysis of the liver indicated lesions with microvesicular steatosis and apoptosis in the hepatic sinusoids [28]. Of note, IFN signaling is not completely disrupted in this STAT1^-/-^ mouse model as that in IFNαβ/γR^-/-^ mice. A partial knockout of STAT1 gene leads to expression of a truncated form of STAT1, which may still be able to mediate a minimal T cell response. As T cells may contribute to LF pathogenesis, the difference in IFN signaling may explain why the STAT1^-/-^ mice are more susceptible to disease than IFNαβ/γR^-/-^ mice [29].

Additionally, the STAT1^-/-^ model is the only available small animal model for SNHL [8]. Both LF2384 and LF2350 clinical isolates from the 2012 Sierra Leone outbreak caused deafness in survivors [8]. Infection with 10^5^ PFU of virus caused permanent hearing loss in all survivors. With a lower dose of virus (10^4^ PFU), hearing loss was present in only 20% of survivors. Histologic examination identified severe damage to the inner ear with significantly fewer outer hair cells; inner hair cells remained intact [8]. Auditory nerves in mice with hearing loss were also damaged, while the nearby facial nerves remained intact. There was significant vacuolization of the spiral ganglion, thinning of the stria vascularis, distention of Reissner’s membrane, and an infiltration of blood cells in the scala tympani. Viral antigen was present in vascular-rich regions, where there was also a remarkable infiltration of CD3+ lymphocytes, indicating an immunopathologic mechanism underlying to the hearing loss [8].

### 3.5. CBA Mice

Intracranial infection of inbred CBA mice with the pathogenic Josiah strain of LASV resulted in disease manifestation, although it was unclear whether the animals were immunocompromised [32]. Infected CBA mice presented with scruffy fur, seizures, weight loss, immobility, severe decubitus paralysis, and death [32]. This route of inoculation allowed for onset of signs of disease between 5 and 7 dpi with 70–100% lethality within 7–12 days [32].

### 3.6. HHD Mice

C57BL/6 mice expressing a human/mouse chimeric HLA-A2.1 instead of the normal MHC class I gene products (humanized HHD mouse model) has recently been established as a novel model for LASV infection [33]. The HHD mouse was most susceptible to infection with the Ba366 strain and featured ~22% lethality and rapid deteriorating post-onset of signs of disease [33]. When infected with 10^6^ PFU of the Ba366 strain, HHD mice began to show ruffled fur and lethargy, as well as elevated concentrations of serum AST, approximately 7–12 dpi [33]. High viral titers were observed in liver, lung, and spleen, whereas lower titers were seen in the kidney [33]. Histology examination indicated severe pneumonitis with signs of pleural effusion, thickening of the interlobular septum, and collapsed alveolar lumen with infiltration of monocytes and macrophages [33]. The liver contained altered cellular distribution, orientation, and shape of its monocytes and macrophages. The spleens also featured disruption of the white and red pulp regions, while monocytes and macrophages were found throughout with scarce T cells.

Depletion of CD4+ T cells, CD8+ T cells, or both resulted in substantial differences in disease manifestations in this model [33]. After infection, the serum AST concentrations remained normal in HHD mice lacking both CD4+ and CD8+ T cells, while the AST concentrations elevated in HHD mice. Additionally, depletion of either CD4+ T cells or CD8+ T cells resulted in partial elevation in AST concentrations [33]. In all groups, similarly high titers of viremia were developed, suggesting T cells had no substantial influence on viremia. C57BL6 mice lacking only CD4+ T cells were able to clear the viral infection, while those lacking only the CD8+ cells featured persistent viremia [33]. MHC-I^-/-^ mice lacking CD8+ T cells confirmed these findings, presenting with no clinical manifestations of disease after infection, despite the high viremia [33]. CD4+ and CD8+ T cell-depleted mice did not demonstrate any significant histological changes in the lung and spleen after infection and also featured limited signs of disease, emphasizing the role of T cells in LASV pathogenesis [33].

In summary, immune-competent laboratory mice are generally resistant to LASV infection, and therefore are not suitable for modeling the severe and fatal LF diseases in humans. Immune-incompetent mice are susceptible to LASV infection but generally develop mild diseases and survive the infection (see Table 2). The chimeric IFNAR^-/-B6^ mice are susceptible to lethal LASV infection but require irradiation of the mice and transplantation with bone marrow progenitor cells from wild type C57BL/6 mice. Recently, progress has been made using the STAT1^-/-^ mice, which recapitulate the pathogenic potency of different LASV isolates and some LF disease signs including hearing loss. This STAT1^-/-^ mouse model may be useful as a small rodent model for vaccine and therapeutic testing.

## 4. Guinea Pig Models

### 4.1. Strain 13 Guinea Pigs

Inbred Strain 13 domesticated guinea pigs have been used as a lethal LF rodent model for studying pathogenesis as well as antiviral, vaccine, and immunotherapy candidates. Unlike their Hartley outbred counterpart, this model does not require virus adaptation [34,35] (Table 3). However, Inbred Strain 13 guinea pigs are not readily available, limiting their use as a LF animal model. In this model at least 90% animals died when infected with at least 2 PFU of LASV (Josiah) [34,36,37]. The clinical isolate NJ2015, from a non-fatal infection, was also non-lethal in Strain 13 guinea pigs [36]. Guinea pigs infected with the Pinneo strain had a 100% survival rate, developing only a mild to moderate disease featuring 5–10% weight loss and lethargy approximately 10 dpi; all animals recovered by 22 dpi [38]. The Z-132 strain was 100% lethal and mimicked a Josiah infection, while the Soromba-R strain was 0–57% lethal with a very mild disease [38,39]. Survivors did not exhibit seroconversion and were susceptible to back challenge [34]. Neutralizing antibodies were not produced [37]. Signs of disease included weight loss, fever, ruffled fur, hunched posture, altered mentation, and conjunctivitis [34,35,36]. Guinea pigs typically became moribund between 10 and 15 dpi [37]. A persistent and high level viremia developed rapidly, typically approximately 5–6 dpi, and peaked at approximately 10 dpi. The highest viral load was identified in the lung, followed by the spleen, pancreas, lymph nodes, adrenal glands, kidneys, salivary glands, liver, and heart [34,35,39]. Histological findings included the presence of viral antigen typically associated with hepatitis, hepatic cell death, hair follicle epithelium cell death, and adrenal cortical necrosis [35]. Laryngitis and heterophilic tracheitis were noted in guinea pigs exposed via aerosol [35]. Interstitial pneumonia was found in all moribund animals with an increase in mononuclear cells, especially macrophages, surrounding the blood vessels and airways [34,35,37,39]. Edema and hemorrhage were also noted in the lungs with an expansion of the alveolar septae [35,39]. Liver samples exhibited a mild to moderate inflammation in portal and sinusoidal regions, vacuolar degeneration, and necrosis [35,37,39]. Half of the guinea pigs developed acute necrotizing nephritis and mild myocarditis or mild to moderate endocarditis and pancarditis [34,35,37]. It is important to note that this damage to the heart is not seen in clinical cases [25]. Adrenal glands featured minimal damage, although they were infected [34,35]. Further analysis of the spleen identified mild heterophilic splenitis [35,39]. The red pulp featured perifollicular to diffuse mononuclear cell proliferation [35]. Most infected guinea pigs developed petechial rashes approximately 12 dpi [35]. Lymphocytolysis and lymphoid depletion was noted in the white pulp of the spleen, thymus, and lymph nodes [35,37]. The pancreas featured acinar cell atrophy as well as other architectural changes [35]. Viral antigen was present in the reproductive tissues of both male and female infected guinea pigs, with males also featuring mild epididymitis [35]. Serum analysis indicated elevated ALT and heterophil concentrations with a transient decrease in platelets and a decrease in blood lymphocytes [35].

Strain 13 guinea pigs presented with conjunctivitis upon LASV infection [36]. Clinically, LASV infections cause conjunctival edema and conjunctivitis in acute infections and transient blindness in survivors. No other animal models have been extensively studied for this aspect of disease, although viral titers have been identified in the aqueous humor of the eye of infected rhesus monkeys [36,44]. The lethal Josiah strain and the non-lethal NJ2015 clinical isolate both induced red and swollen conjunctiva with associated ocular discharge in the animals [36]. Viral antigen was detected in the eyes of all infected guinea pigs achieving humane euthanasia criteria, while none was detected in those of survivors [36]. Survivors presented with minimal to mild lymphocytic inflammation; lethal infections all presented with mild to moderate inflammation [36].

### 4.2. Hartley Guinea Pigs

Outbred Hartley domesticated guinea pigs are commercially available and susceptible to infection with the widely used Josiah strain of LASV (Table 3). However, the Josiah strain caused lethal infection in only 30–67% of animals regardless of the infection dose. Survivors seroconverted and were not susceptible to back challenge [34]. To achieve uniform lethality, the Josiah strain must be adapted in guinea pigs through serial passage [25,34,35,40,41]. Guinea pigs infected with the guinea pig-adapted LASV (GPA-LASV) developed a fever approximately 6–9 dpi as well as respiratory distress and hypothermia [41,42]. Weight loss was rapid and variable, ranging from 8% to over 20%, which is typical humane euthanasia criteria [40,41,42]. Other clinical manifestations included an unstable gait, a noticeable lack of grooming with associated rough/ruffled fur, delayed responsiveness/lethargy, recumbency, and a reddening of the footpads and ears [40,41]. Death occurred by an average of 15 dpi [41]. Viral titers were observed in the serum, spleen, liver, and lung [41,42]. Histologic analysis of the liver indicated lymphohistiocytic hepatitis and hepatocellular degeneration [41]. Spleen samples contained sinus histiocytosis and lung samples featured interstitial pneumonia [41]. The GPA-LASV/Hartley guinea pig model has been often used in development of antivirals and vaccine against LF [40,41,42].

Recently, new advances have been made in the development of the outbred LASV-infected Hartley guinea pig model by using the LF2384 clinical isolate. Uniform lethality could be achieved in outbred Hartley guinea pigs infected by the LF2384 clinical isolate without species-specific adaptation, at a dose as low as 100 PFU [45]. LF2384-infected guinea pigs presented with signs of disease such as fever, hypothermia before death, as well as weight loss by 10 dpi, scruffy coats by 12 dpi, and loss of appetite as well as lethargy by 13 dpi [45]. Neurological disease signs were not observed in this model [45]. Viral loads were detected in the liver, kidney, spleen, lung, and brain, but were not detectable in survivors of a low dose infection [45]. Guinea pigs succumbing to disease presented with thrombocytopenia, neutropenia, and lymphopenia [45]. Gross pathology indicated small spleens [45]. While this model requires further characterization, the advantages of this new LF model include the use of clinical isolates directly from patients without adaptation and the commercial availability of the animals. Additionally, the outbred background may allow for mimicking infections in humans with diverse genetic backgrounds. The newly developed LF2384 LASV/Hartley guinea pig model will be useful for vaccine and therapeutic development, which is demonstrated in a recent study showing that immunization with an adenovector-based LASV vaccine candidate efficaciously protected animals from lethal LASV infection [43].

## 5. Non-Human Primate Models

### 5.1. Squirrel Monkey Model

Squirrel monkeys (*Saimiri sciureus*) infected with the Bah strain of LASV developed variable disease. One out of four animals became moribund [46] (Table 4). These primates develop depression, tremors, drooling, anorexia, lassitude, and polydipsia as signs of disease. Serologic analysis indicated persistent viremia with no antibody development at 28 dpi [46]. High viral titers were detected primarily in the liver, kidney, and lymph nodes, while lower virus loads were found in the spleen, brain, liver, kidneys, adrenal gland, urine and heart [25,46]. The presence of the virus was associated with significant tissue necrosis [25]. Spleen and lymph node samples featured necrosis in the germinal centers; spleen samples and adrenal glands also contain regions of focal hemorrhage [46]. While the liver suffered from hepatitis with fatty metamorphosis and an infiltration of leukocytes and mononuclear cells, hepatocytic regeneration was noted [46]. Renal tubular necrosis and regeneration was present in the kidney, while heart samples featured severe myocarditis with vacuolar degeneration and necrosis [46]. Some animals developed neuropathology with chronic inflammation of the meninges, a significant infiltration of mononuclear cells and lymphocytes into the pancreas with acute arteritis, or minor pathologic changes in the adrenal gland, prostate, and bone marrow [46].

### 5.2. Marmoset Model

The common marmoset (*Callithrix jacchus*) has been identified as a good model for Lassa fever (Table 4). Primates infected with the Josiah strain of LASV develop a systemic disease featuring low fever and rapid weight loss [47]. These monkeys also exhibited behavioral changes, such as depression and anorexia, as well as reduced stool production [47]. Severe morbidity occurred between 15 and 20 dpi [47]. Serum analysis indicated an elevation in AST, ALT, and alkaline phosphatase concentrations as well as a decrease in albumin and platelet concentrations [47]. High viral titers were observed in the liver, spleen, and lymph nodes [47]. Livers were enlarged with pale patches. Histologic analysis indicated multifocal hepatic necrosis with mild inflammation [47]. Spleens were also enlarged with mild to moderate lymphoid depletion [47]. Likewise, lymph nodes were enlarged with lymphocyte necrosis and an infiltration of inflammatory cells [47]. Kidneys were pale with interstitial nephritis [47]. Lung samples featured hemorrhage present in most lobes with mild to moderate multifocal interstitial pneumonitis, septal thickening, and multifocal edema [47]. These samples also had increased infiltration of lymphocytes and macrophages [47]. The adrenal cortex also featured mild to moderate inflammation and multifocal necrosis [47].

### 5.3. Rhesus Monkey (Rhesus Macaque) Model

Rhesus monkeys (*Rhesus macaques*) infected with the Josiah strain of LASV develop severe disease with prolonged viremia [44]. Succumbed animals developed disease signs by 7 dpi, including severe petechial rash, hiccups, lethargy, aphagia, huddled posture, constipation, conjunctivitis, anorexia, weight loss, decreased water intake/dehydration, facial and periorbital edema, bleeding from the gums and nares, cough, and a slight fever [44,48,49,54,55]. Fever persisted until death or a sudden hypothermia happened just before death approximately 10–14 dpi [44,49,54,55]. Subcutaneous inoculation with 10^6.1^ PFU of Josiah strain of LASV proved to be lethal in 60% of the animals [47]. Serologic analysis indicated antibody production by 10–12 dpi, although this was not correlated with viral clearance and animal recovery [44,49]. Elevated AST, ALT, and blood urea nitrogen (BUN) with a transient and moderate leukopenia was also noted [49,54,55]. Hematocrit, hemoglobin, fibronectin, and red blood cell counts decreased; platelet counts trended toward a decrease, although remained within the normal range [54,55]. Viremia appeared typically by 4–5 dpi with titers over 10^4^ PFU/mL in lethally infected monkeys, significantly higher than those in survivors [49,54,55]. Viral loads were found in every organ tested including the adrenal glands, liver, lung, pancreas, brain, bone marrow, kidney, lymph nodes, spleen, muscle, heart, thymus, testis, salivary gland, urine, CSF, and intestines [44,49,55]. The highest titers were in the liver, spleen, and adrenal glands [55].

Gross pathological analysis indicated scattered petechial and visceral hemorrhage along with the presence of mild to moderate pleural effusions [44,48]. Liver and adrenal gland tissues presented with necrosis, with markers of regeneration of hepatocytes and a slight infiltration of inflammatory cells [44,48,49,55]. Interstitial pneumonia with edema, thickened alveolar septae, and pulmonary arteritis were present in the lung [44,48,49,55]. Spleen samples indicated lymphocytopenia and presence of viral antigens only in the red pulp [48,49]. Infected primates also developed mild to moderate interstitial and perivascular myocarditis and pericardial edema. Severe meningoencephalitis with significant perivascular cuffing was noted, albeit rarely [44,48]. Infiltration of erythrocytes and macrophages was noted in the small intestine. Lesions and a multifocal cortical interstitial mononuclear infiltrate were noted in the kidney [48,55]. Seventy-eight percent of infected primates developed lesions in the CNS with mild lymphocytic cuffing of the vessels of the brain, spinal cord, and meninges [48]. Twenty percent of primates suffered lymphocytic infiltration of the spiral ganglia; mild choriodoretinitis was also noted [48]. The arterial lesions, vasculitis, meningoencephalomyelitis and skeletal myositis observed in this monkey model were rarely, if at all, noted in human LF cases [44].

### 5.4. Cynomolgus Macaque Model

Josiah- or Z-132-infected “crab-eating” (cynomolgus) macaques developed a severe hemorrhagic disease with up to 100% of primates becoming moribund or dying between 11 and 18 dpi [38,39,50,51,52,53,56]. A study with the Soromba-R strain found two of three animals died (66% lethality) after infection and a prolonged time to euthanasia [39]. Infection with the Liberian LASV Z-132 strain was 100% lethal by 18 dpi [35]. Infection with 10^3^ FFU AV strain featured only 67% lethality. All animals survived infection with 10^7^ FFU of the AV strain, probably due to a high concentration of defective interfering (DI) viral particles in the higher dose [57]. Clinical signs, such as fever, weight loss, lethargy, dull appearance, reluctance to move/hypoactivity, anorexia, rashes, facial edema, hunched posture, ruffled fur, piloerection, bleeding from puncture sites, dehydration, epistaxis, acute respiratory syndrome, and mild to moderate depression appeared approximately 5-7 dpi, but might begin as early as 3 dpi [38,39,50,51,52,56,57,58]. Fever spiked between 7 and 10 dpi [39]. Viremia might start as early as 3–4 dpi, but was usually detectable 6–10 dpi, peaked 12–14 dpi, and continued to maintain high titers (up to 10^7^ PFU/mL) until death or euthanasia [39,50,51,53,56,58]. Onset of fever was associated with detectable viremia. Survivors typically had a lower virus titer and cleared the virus by 28 dpi [50,57]. Survivors suffered neurological disorders such as tremors, reduced appetite, ataxia, convulsions/seizures, and unilateral or bilateral deafness [50,52,56,58]. Sixty-seven percent of the survivors developed hearing loss, which was thought to be driven by an immune-associated systemic vasculitis [50,56]. This hearing loss was present in all survivors of one study, presenting as unilateral or bilateral hearing loss [56].

Serologic analysis indicated elevated concentrations of AST, ALT, alkaline phosphatase (ALP), gamma-glutamyltransferase (GGT), total bilirubin (TBIL), and BUN as well as decreased concentrations of albumin, total protein, and amylase [38,39,50,51,58]. Transient lymphopenia, monocytopenia, neutropenia, and eosinopenia were also noticed at 4–10 dpi [38,39,50,51,58]. Platelet concentrations typically tended to decrease but remained within normal ranges, along with reduced hemoglobin, hematocrit, and creatinine concentrations [39,50,51,58]. The autoimmune markers C-reactive protein, antineutrophil cytoplasmic antibodies, and circulating immune complexes were also elevated in survivors with hearing loss [56]. Neutralizing antibodies were not detected in primates that succumbed to the infection but developed slowly in survivors approximately 14 dpi [50,56,57]. An early and strong T cell response was noticed in survivors [57]. Viral titers were detected at high levels in the spleen, lung, and liver, and at lower levels in the lymph nodes, kidney, and brain by 7 dpi [38,39,51,56,57,58].

Gross pathologic analysis revealed enlarged livers, spleens, adrenal glands, pancreas and lymph nodes, along with slight lung discoloration and pericardial effusion [38,39,57,58]. Lung samples featured mild to severe interstitial pneumonia with thickening of the alveolar septae [38,57,58]. Interestingly, although Soromba-R infections presented with a significantly less severe disease, the pathology of the lung was remarkable; 50–100% of lobes were reddened with lesions and extensive pulmonary infiltrations [38]. Liver samples were pale yellow and friable, featuring mild and multifocal portal hepatitis with random regions of necrosis [38,50,58]. Brain samples presented with meningoencephalitis in the frontal lobe, brainstem, and cerebellum with lesions. Neuritis was noted in the optic nerve [38,58]. Both lymph nodes and the white pulp of the spleen suffered follicular hyperplasia and mild lymphocytolysis. Spleens were also friable with fibrinous deposits in the red pulp [38,57,58]. Kidney samples appeared congested with mononuclear cell infiltrates [57,58]. Petechial hemorrhage and necrosis were noted on the bladder, while the ovaries and uterus were inflamed [57,58]. The thymus featured marked atrophy; myocarditis and necrotizing coronary arteritis were noted in the heart [58].

## 6. Surrogate Models of Lassa Fever

### 6.1. Pichindé Virus in Guinea Pigs

Pichindé virus (PICV) is a non-pathogenic, New World mammarenavirus isolated from cricetine rodents (*Oryzomys albigularis*) in Colombia, South America [59,60]. PICV infection of Strain 13 guinea pigs can cause Lassa-like disease and therefore has been used as a surrogate LASV model in BSL2 labs (Table 5) [61,62,63]. A minimum of four sequential passages of PICV in guinea pigs was required to result in 100% lethality [63,64]. Infected guinea pigs were hypoactive and lethargic, with ruffled fur, decreased food/water intake, rapid and shallow breathing, slobbering, and significant weight loss before becoming moribund [62,64]. Viremia appeared by 2 dpi and steadily increased until death at 16 dpi [62,63,64]. Infected guinea pigs featured extreme leukopenia beginning approximately 13 dpi and a transient neutrophilia [63]. Serologic findings included impaired platelet function, decreased activity of coagulation factors, and thrombocytopenia, decreased white blood cell concentrations and increased hematocrit [64]. AST concentrations were also elevated, starting from approximately 9 dpi [63]. Histologic analysis indicated pathological lesions in the liver, spleen, pancreas, lungs and gastrointestinal tract [61,62,63,64]. Scattered necrotic regions were found in lymphoid tissue and the bone marrow [64].

Unlike Strain 13 guinea pigs, Hartley outbred guinea pigs did not uniformly succumb to infection by PICV CoAn 4763 strain [63,65]. The lethality was approximately 43% when animals were infected with 3000 PFU PICV CoAn 4763 [63]. Virus was cleared by 16 dpi in survivors [63]. When the CoAn 4763 strain passaged in Strain 13 guinea pigs for 18 passages, the resulting P18 strain was more virulent than the P2 strain, which was passaged only twice [65,66,67]. Infection with 100 PFU of the PICV P18 strain caused weight loss, fever and uniformly lethal infection in outbred guinea pigs [65,66,67]. Virus was present in almost all organs, with the highest titers in the adrenal glands, lungs, stomachs and livers [66]. Lower yet still notable titers were found in the brain [66]. P2-infected guinea pigs did not present with any detectable viral titer in any tested organs [66]. Additionally, P18 PICV-infected guinea pigs presented with a longer and more severe disease than their P2-infected counterparts [66]. The level of viremia was correlated with disease severity [66].

### 6.2. Pichindé Virus in Golden Hamsters

Pichindé virus infection also caused Lassa-like disease in Golden hamsters (*Mesocricetus auratus*) with various lethality depending on the virus strains (Table 5) [68]. The lethality could be 100% in new-born LVG/Lak outbred hamsters, and mortality was uncommon after 8 days post-birth. Peak viral titers in adults reached as high as 10^3^ PFU/mL. MHA/Lak inbred hamsters demonstrated 100% lethality with as low as 35 PFU of virus regardless of ages [68]. Adults at 8 dpi developed viral titers over 10^8^ PFU/mL. Both hamster models produced antibodies against the virus. Spleen, liver, and kidneys were the primary affected organs [68].

### 6.3. Pirital Virus in Golden Hamsters

Pirital virus, a non-human pathogenic New World mammarenavirus isolated from western Venezuela, causes a Lassa-like disease in Golden Hamsters [69]. These hamsters developed a severe disease and died approximately 7 dpi [69]. Interstitial pneumonia, multifocal hepatic necrosis, as well as splenic lymphoid depletion and necrosis were noted upon histologic analysis [69]. The brain, kidneys, and intestine were not significantly affected and there was no significant infiltration of inflammatory cells in the affected tissues [69]. Some of the infected hamsters presented with hemorrhagic signs from the mouth or puncture wounds. The clotting deficiency was noted in the collected blood samples, in which clotting rates were either non-existent or extremely slow [69].

### 6.4. Lymphocytic Choriomeningitis Virus (LCMV) in Rhesus Monkeys

The virulent WE strain of lymphocytic choriomeningitis virus (LCMV), a pathogenic Old World mammarenavirus, causes a uniformly lethal Lassa-like disease in rhesus monkeys infected intravenously (Table 5) [70]. Viremia was detected beginning at 4 dpi [70]. The liver especially was affected in this model, with significant gene expression changes noted [70,71]. However, of five primates infected intragastrically (IG), lethality only reached 20%, with only two of the monkeys becoming viremic [72]. Signs of disease included weight loss and serological analysis indicated elevated ALT and AST as well as thrombocytopenia, transient neutrophilia [72].

## 7. Summary

Animal models are essential for pathogenesis studies and LASV vaccine and antiviral developments. An ideal LF animal model should match the disease manifestation and progression, pathological changes, and the various pathogenicity of different strains in humans. Additionally, outbred animals with diverse genetic background have an advantage of mimicking infection in human populations. Other important factors include the immune-competency, the availability and the cost of the animals. The prototypic Josiah strain has been the most commonly used LASV strain in laboratory studies. However, the genetic diversity of LASV should also be considered when developing LF animal models.

The NHP models (Table 4), particularly “crab-eating” (cynomolgus) macaques, develop a disease very similar to clinical cases of LF and thus are excellent models for vaccine and antiviral evaluations as well as pathogenesis studies. However, the use of NHPs is extremely limited due to their relatively high cost, the difficulty of handling the animals, as well as the requirements for experienced staff and adequate space in high containment BSL4 facilities. Additionally, it is not easily feasible to perform NHP studies using large group sizes, making statistical significance difficult to ascertain.

Inbred Strain 13 guinea pigs are useful model animals, but the animals are not readily available and lack genetic diversity. Outbred Hartley guinea pigs are commercially available. However, the widely used Josiah strain must be adapted in guinea pigs in order to cause LF-like disease. PICV infection of Hartley guinea pigs can be used as a surrogate animal model of LF. Using the clinical LASV LF2384 isolate, which was isolated from a fatal case and has experienced minimal passaging in the laboratory, uniformly lethality can be achieved in Hartley guinea pigs without species-specific adaptation. Although further characterization is required, this new model shows much potential as a relevant and relatively inexpensive rodent model that combines the advantages of diverse genetic background, immunocompetency as well as the convenience of the animal’s small size and large numbers that can be used in an experiment, which can ensure the rigor and reproducibility of the studies. However, one limitation of using guinea pigs is the lack of available laboratory reagents and a collection of genetically modified animals for immunological and related studies. Nevertheless, guinea pig models could be ideal for vaccine and antiviral screening and potentially pathogenesis studies. A summary of guinea pig LF disease manifestations and pathologies can be found in Table 3.

Immune-competent laboratory mice are generally resistant to LASV infection (Table 2). The STAT1^-/-^ mouse model is the only known animal model for studying LASV-associated hearing loss. This new mouse model has been used in studies on the pathogenesis and immunology of LF, which indicate an immunopathological mechanism of LF. This mouse model may be useful in initial vaccine and antiviral drug screening, as it is a lethal model of infection. However, its immune-incompetency should be considered, as deficiency in interferon signaling may have negative effects on such studies [73,74]. 

With the increased fatality of LF in the most recent 2018–2019 outbreaks, there is an urgent need for the rapid development of vaccines and antivirals. Development of small animal models that resemble human disease is crucial to facilitate vaccine and therapeutic testing. The best option at the current stage seems to be performing initial screening in lethal rodent models, such as the aforementioned STAT1^-/-^ mouse model and the newly developed LF2384/outbred Hartley guinea pig model, followed by validation of promising candidates in NHPs. Additionally, the STAT1^-/-^ model is useful as a new platform for investigating the mechanism of LF-associated hearing loss, which affects approximately one third of survivors.

## Figures and Tables

**Table 1:** Information for Lassa virus (LASV) strains examined in animal models *.

Strain	Lineage	Lethal/Fatal	Host	Isolation Country	Isolation Year	Reference
Josiah	IV	Yes	Human	Sierra Leone	1976	[6]
AV	V	Yes	Human	Ghana/Ivory Coast	2000	[6]
Ba366	IV	No	*Mastomys* *natalensis*	Guinea	2003	[6]
LF2384	IV	Yes	Human	Sierra Leone	2012	[6,8]
LF2450	IV	No	Human	Sierra Leone	2012	[6,8]
Soromba-R	V	No	*Mastomys* *natalensis*	Soromba, Mali	2009	[6]
Pinneo	I	No	Human	Lassa, Nigeria	1969	[6]
NJ2015	IV	Yes	Human	Liberia	2015	[9]

* Only strains mentioned in this review that have known isolation information are listed in this table.

**Table 2:** Mouse models of LASV infection.

Mouse	Virus Strain	Max. Dose	Route	Lethality	Signs of Disease	Affected Organs	Reference
Natal mastomys	Unknown	Not provided	IP ^d^	No	asymptomatic, persistent infection, virus shed in saliva and urine	lung, spleen, liver, kidney, brain, bladder, lymph nodes	[25]
IFNAR^-/-^	Josiah, AV, BA366, Nig04-10	10^5^ PFU	IV ^e^	No	persistent viremia, bodyweight loss, no fever and neurological signs	lung, liver, spleen brain, kidney, heart	[27,28,29,30]
Chimeric IFNAR^-/-B6^	Ba366, AV, Ba289, Nig04-10, Nig-CSF	10^3^ FFU	IP	100%	liver damage, vascular leakage, systemic viral dissemination, weight loss, hypothermia, elevated AST/ALT ratio	liver, lung, spleen, kidney, heart, brain	[29,31]
IFNαβ/γR^-/-^	Josiah, LF2384 ^a^ LF2450 ^b^	10^5^ PFU	IP	No	minor and transient weight loss, clearance of virus by 25 dpi, no hearing loss	spleen, lung, liver, brain, kidney	[8,30]
STAT1^-/-^	Josiah	10^4^ PFU	IP	100%	weight loss, systematic infection	spleen, lung, liver, brain, kidney	[28]
	LF2384 ^a^ (fatal)	10^5^ PFU	IP	80%	fever, high level viremia, weight loss, increased ALT level, decreased albumin, WBC, and monocyte counts, hearing loss associated with infiltration of CD3^+^ lymphocytes	brain, liver, spleen, lung, kidney, heart	[8,28]
	LF2450 ^b^ (non-fatal)	10^5^ PFU	IP	0–50%	hearing loss	brain, liver, spleen, lung, kidney, heart	[8]
CBA	Josiah	10^3^ PFU	IC ^f^	70–100%	scruffy fur, seizures, weight loss, immobility, and severe decubitus paralysis	not stated	[32]
HHD	Ba366 ^C^	10^6^ PFU	IV	22%	ruffled fur, lethargy, elevated AST level, high level viremia, severe pneumonitis	liver, lung, spleen, kidney	[33]

Note: ^a^ LF2384: clinical isolate from a lethally infected patient; ^b^ LF2450: clinical isolate from a survivor; ^C^ isolate from *Natal mastomys*; PFU, plaque forming unit; ^d^ IP: intraperitoneal; ^e^ IV: intravenous; ^f^ IC: intracranial.

**Table 3:** Guinea pig models of LASV infection.

Guinea Pig	Strain	Max. Dose	Route	Lethality	Signs of Disease	Affected Organs	Refences
Strain 13 (inbred)	Josiah	> 2 PFU10^4^ TCID_50_	SC ^c^ IP	>90%	weight loss, fever, ruffled fur, hunched posture, conjunctivitis, hepatitis, interstitial pneumonia, edema and hemorrhage in lungs	lung, spleen, pancreas, lymph nodes, adrenal and salivary glands, kidneys, liver, heart	[34,36,37]
	Z-132	10^4^ TCID_50_	IP	100%	Josiah-like	lung, liver, spleen	[38,39]
	Soromba-R	10^4^ TCID_50_	IP	0–57%	survivors only show minor weight loss	liver, lung, spleen	[28,34,35,38,39]
	Pinneo	10^4^ TCID_50_	IP	No	mild to moderate disease	N.A. *	[38]
	NJ2015	10^4^ FFU	SC	No	weight loss, fever, red and swollen conjunctiva	eye (focus of the study)	[36]
Hartley (outbred)	Josiah	>2 PFU10^3^ PFU	SCIP	30–67%	inapparent infection in survivors	N.A. *	[25,34,35,40,41]
	GPA-Josiah ^a^	10^3^ TCID_50_	IP	100%	weight loss, fever, lethargy, respiratory distress, hypothermia	spleen, liver, lung	[25,34,35,40,41,42]
	LF2384 ^b^	10^4^ PFU	IP	100%	fever, weight loss, hypothermia, lethargy, thrombocytopenia, neutropenia, and lymphopenia	liver, kidney, spleen, lung, brain	[43]

* N.A: not available; ^a^ GPA-Josiah: guinea pig-adapted LASV Josiah strain; ^b^ LF2384: clinical isolate from a fatally infected LF patient; ^c^ SC: subcutaneous

**Table 4:** Non-human primate (NHP) models of LASV infection ^a^.

NHP	LASV Strain	Max. Dose	Route	Lethality	Signs of Disease	Affected Organs	Reference
squirrel monkeys	Bah	10^6.8^ TCID_50_	IM ^b^	25%	depression, tremors, drooling, anorexia, lassitude, polydipsia	liver, kidney, lymph nodes, spleen, brain, adrenal gland, heart	[46]
marmoset	Josiah	10^6^ PFU	SC	100%	low fever, rapid weight loss, depression, anorexia, elevated concentrations of AST, ALT, alkaline phosphatase, decreased concentrations of albumin and platelet	liver, spleen, lymph nodes, kidney, lung, adrenal gland	[47]
rhesus monkey	Josiah	10^6.1^ PFU	SC	50–60%	severe petechial rash, hiccups, lethargy, aphagia, huddled posture, constipation, conjunctivitis, anorexia, weight loss, decreased water intake/dehydration, facial and periorbital edema, bleeding from the gums and nares, cough, fever	adrenal glands, liver, lung, pancreas, brain, bone marrow, kidney, lymph nodes, spleen, muscle, heart, thymus, testis, salivary gland, CSF, intestines	[44,48,49]
“crab-eating” cynomolgus macaques	Josiah	10^4^ PFU	IM	Up to 100%	fever, weight loss, lethargy, dull appearance, reluctance to move/hypoactivity, anorexia, rashes, facial edema, hunched posture, ruffled fur, piloerection, bleeding from puncture sites, dehydration, epistaxis, acute respiratory syndrome, neurological signs including deafness	lymph nodes, spleen, liver, reproductive organs, kidney, lung, heart, CNS	[38,39,50,51,52,53]
	Z-132	10^4^ TCID_50_	IM	100%	Josiah-like	spleen, liver, lung	[38,39]
	Soromba-R	10^4^ TCID_50_	IM	66%	less severe than Josiah strain infection, moderate to severe pulmonary lesions	similar to Josiah and Z-132	[39]

^a^ Another NHP model, for which the literature was not available to the authors, is the hydramas baboon model; ^b^ IM: intramuscular.

**Table 5:** Surrogate models of LASV infection.

Animal	Virus/Strain	Max. Dose	Route	Lethality	Signs of Disease	Affected Organs	Reference
Strain 13 (inbred) guinea pig	Pichindé virus	>3 PFU	SC	100%	hypoactivity, lethargy, ruffled fur, decreased appetite, rapid/shallow breathing, slobbering, weight loss, leukopenia, and transient neutrophilia	liver, spleen, pancreas, lung, gastrointestinal tract, lymphoid tissue, bone marrow	[61,62,63,64]
Hartley (outbred) guinea pig	Pichindé virus/P18	100 PFU	IP	100%	weight loss and fever	adrenal glands, lung, stomach, liver, brain, heart, spleen, pancreas, intestine, kidney, lymph node	[65,67]
	Pichindé virus/P2	10^4^ PFU	IP	<100%	avirulent	none	[66]
	Pichindé virus/CoAn 4783	3000 PFU	IP	43%	weight loss and fever	cleared in survivors	[65]
LVG/Lak outbred golden hamsters	Pichindé virus	500 PFU	SC	0–100%	lethal infection up to 8 days post-birth; uncommon afterwards	spleen, liver, kidney	[68]
MHA/Lak inbred golden hamsters	Pichindé virus	3.5 × 10^6^ PFU	IP	100%	lethal infection regardless of age	spleen, liver, kidney	[68]
Golden hamsters	Pirital virus	10^5^ TCID_50_	IP	>50%	hemorrhage, interstitial pneumonia, multifocal hepatic necrosis, splenic lymphoid depletion and necrosis	lung, liver, spleen	[69]
Rhesus monkeys	LCMV-WE	10^3^ PFU	IV	100%	not listed	not listed	[70,71]
		10^8^ PFU	IG ^a^	20%	weight loss, elevated AST and ALT, thrombocytopenia, transient neutrophilia	liver	[72]

^a^ IG: intragastic
